# The causal relationship between immune cells and different kidney diseases: A Mendelian randomization study

**DOI:** 10.1515/med-2023-0877

**Published:** 2023-12-20

**Authors:** Lei Pang, Zijun Ding, Hongqiang Chai, Weibing Shuang

**Affiliations:** Department of Urology, The Fifth Hospital of Shanxi Medical University (Shanxi Provincial People's Hospital), Taiyuan City, 030012, Shanxi Province, China; The First Clinical Medical College of Shanxi Medical University, No. 85, Jiefang South Road, Yingze District, Taiyuan City, 030012, Shanxi Province, China; Department of Neonatology, Shanxi Children's Hospital, Taiyuan City, 030013, Shanxi Province, China; Department of Urology, The First Hospital of Shanxi Medical University, No. 85, Jiefang South Road, Yingze District, Taiyuan City, 030012, Shanxi Province, China; The First Clinical Medical College of Shanxi Medical University, Taiyuan City, 030012, Shanxi Province, China

**Keywords:** neutrophil, basophil, lymphocyte, eosinophil, macrophages, kidney disease, Mendelian randomization

## Abstract

Studies have suggested that the progress of most kidney diseases from occurrence to course and subsequent related complications are closely related to inflammatory reaction. Increased common leukocytes count in the family (neutrophils, eosinophils, basophils, lymphocytes, etc.) are also involved in the tissue damage of kidney diseases. However, these studies are only traditional observational studies, which cannot prove whether there is a causal relationship between these four kinds of leukocytes count and kidney diseases. We aim to explore the causal relationship between these four kinds of leukocytes count and kidney diseases by Mendelian randomization (MR). Large sample size of the genome-wide association database of four cell traits (neutrophil, basophil, lymphocyte, and eosinophil cell counts) in the leukocyte family were used as exposure variables. The outcome variables were various renal diseases (including chronic renal failure, acute renal failure, hypertensive heart or/and kidney disease, hypertensive renal disease, disorders resulting from impaired renal tubular function, and type 1 diabetes with renal complications). The covariates used in multivariable MR are also four cell traits related to blood cells (neutrophil, basophil, lymphocyte, and eosinophil cell counts). Instrumental variables and single nucleotide polymorphic loci were identified (*P* < 5 × 10^−8^. Linkage disequilibrium *R*^2^ < 0.001). The causal relationships were studied by inverse variance weighted (IVW), weighted median, and MR-Egger regression. Sensitivity analysis was also performed. In our study, IVW analysis results showed that increased neutrophil cell count was a risk factor for chronic renal failure (OR = 2.0245861, 95% CI = 1.1231207–3.649606, *P* = 0.01896524), increased basophil cell count was a risk factor for chronic renal failure (OR = 3.975935, 95% CI = 1.4871198–10.62998, *P* = 0.005942755). Basophil cell count was not a risk factor for acute renal failure (OR = 1.160434, 95% CI = 0.9455132–1.424207, *P* = 0.15448828). Increased basophil cell count was a protective factor for hypertensive heart and/or renal disease (OR = 0.7716065, 95% CI = 0.6484979–0.9180856, *P* = 0.003458707). Increased basophil cell count was a risk factor for disorders resulting from impaired renal tubular function (OR = 1.648131, 95% CI = 1.010116–2.689133, *P* = 0.04546835). Increased lymphocyte cell count was a risk factor for hypertensive renal disease (OR = 1.372961, 95% CI = 1.0189772–1.849915, *P* = 0.03719874). Increased eosinophil cell count was a risk factor for type 1 diabetes with renal complications (OR = 1.516454, 95% CI = 1.1826453–1.944482, *P* = 0.001028964). Macrophage inflammatory protein 1b levels was a protective factor for renal failure (OR = 0.9381862, 95% CI = 0.8860402–0.9934013, *P* = 0.02874872). After multivariable MR was used to correct covariates (neutrophil, basophil, and lymphocyte cell counts), the correlation effect between increased eosinophil cell counts and type 1 diabetes with renal complications was still statistically significant (*P* = 0.02201152). After adjusting covariates (neutrophil, basophil, and eosinophil cell counts) with multivariable MR, the correlation effect between increased lymphocyte cell counts and hypertensive renal disease was still statistically significant (*P* = 0.02050226). This study shows that increased basophils can increase the relative risk of chronic renal failure and renal tubular dysfunction, and reduce the risk of hypertensive heart disease and/or hypertensive nephropathy, while increased basophil cell count will not increase the relative risk of acute renal failure, increased neutrophil cell count can increase the risk of chronic renal failure, increased lymphocyte cell count can increase the relative risk of hypertensive nephropathy, and increased eosinophil cell count can increase the relative risk of type 1 diabetes with renal complications. Macrophage inflammatory protein 1b levels was a protective factor for renal failure.

## 1 Introduction

According to the data from the American Society of Nephrology, the European Renal Society-European Dialysis and Transplantation Society, and the International Society of Nephrology, at least 850 million people worldwide are suffering from kidney diseases. In recent years, kidney diseases have gradually separated from the impression of the so-called “geriatrics,” and the trend of youth is becoming increasingly obvious [1]. There are many clinical classifications of kidney diseases, including acute renal failure, chronic renal failure, hypertensive renal disease, diabetes with renal complications, and kidney disease caused by renal tubular dysfunction. All of the above diseases can lead to renal failure. Renal failure is a slow decline in renal function based on various chronic kidney diseases. With the progress of the disease, end-stage renal disease (ESRD) is gradually formed [2]. Tracing back to the origin of the basic disease of renal failure, chronic glomerulonephritis is the most common primary disease, and the pathogenesis of chronic glomerulonephritis is mainly immune-mediated inflammatory damage, that is, immune complexes stimulate monocytes to produce interleukin (IL)-1, IL-6, TNF- α and other inflammatory cytokines, causing inflammatory pathological damage to the kidney [3]. Secondary kidney disease is common in hypertensive nephropathy and diabetic nephropathy. Clinical observation has confirmed the inflammatory indicators C-reactive protein (CRP) and TNF- α. The level is significantly increased in patients with hypertensive nephropathy and diabetes nephropathy, so in essence, hypertensive nephropathy and diabetes nephropathy are inflammatory diseases caused by metabolic disorder [4]. With the progression of chronic renal failure, the level of CRP, and TNF-α in patients’ serum are showing an up ward trend. The content of inflammatory biomarkers such as IL-6 will increase significantly, so even if an exogenous infection is not combined, chronic renal failure disease itself can cause the inflammatory reaction in the body [5]. These findings suggest that the progress of most kidney diseases from the occurrence to the course of the disease and the subsequent occurrence of related complications are closely related to the inflammatory reaction.

The common cells in immune cells include neutrophils, eosinophils, basophils, lymphocytes, macrophages, etc. The role of immune cells in the body’s resistance to infection and removal of foreign bodies has long been recognized. However, in recent years, studies have found that the increase in the number of immune cells is also involved in the tissue damage in kidney diseases [6], but these studies are only traditional observational studies, it cannot be proven whether there is a causal relationship between different immune cells and kidney disease. We have conducted research on this issue.

In traditional observational studies, potential confounding and reverse causality will affect their causal inference ability [7]. Mendelian randomization (MR) is a type of instrumental variable (IV) analysis, which uses genetic variation as IV to detect and quantify causality [8]. In recent years, the MR research method has been more and more widely used in observational research because it can overcome the influence of potential confounding and reverse causality [9]. Early MR method research is usually conducted in a small sample population, and only a small amount of genetic variation is used [10], which makes the effectiveness of MR research low. However, with the discovery of a large number of genetic variations closely related to specific traits in the biological community, and the public release of hundreds of thousands of data on the relationship between exposure and disease and genetic variation [11] by many large samples of genome-wide association studies (GWAS) [12], a revolution has taken place in this field. These aggregated data enable researchers to estimate the genetic association in large sample data, thus promoting the development of MR research. In recent years, the methodology in this field has also been rapidly updated. The new method overcomes some specific limitations of the traditional MR method [13]. This study uses univariate and multivariate MR methods to analyze the summary data of the GWAS, and explores the causal relationship between different immune cells and different kidney diseases.

### 1.1 IV analysis and MR hypothesis

MR is essentially consistent with the basic assumption of IVs, but because MR regards genetic variation as IV, it has some particularity in terms. Hypothesis 1: The generic variables are related to the exposure factors of interest. This hypothesis is expressed in MR as a genetic variation associated with (non-genetic) exposure of interest. In this hypothesis, the correlation between genetic variants and exposure factors is not necessarily a causal relationship. It should be noted that when IV analysis is used in actual research, genetic variants need to have a strong correlation with exposure factors. When this correlation is weak, generic variants are called “weak instruments.” These weak instruments will cause great limitations to MR research. Hypothesis 2: Genetic variants are independent of confounders between exposure factors and outcome factors. Genotypes in MR should not be associated with confounding factors in the exposure–outcome relationship. Although this assumption is often difficult to prove directly, it can sometimes be falsified by comparing the variation of the exposure–outcome relationship with the relationship between known confounding factors [14]. Hypothesis 3: Generic variables have no direct impact on outcome factors, but only affect the results by exposing generic variables [15]. This hypothesis is also called the exclusion limit criterion and the null hypothesis in MR. In the past, it was difficult to prove this hypothesis in research, but some methods developed in recent years can detect its existence and unbiasedly estimate the causal effect of exposure and outcome in violation of this hypothesis [16].

## 2 Data and methods

### 2.1 Data source and research design

**Figure 1:**
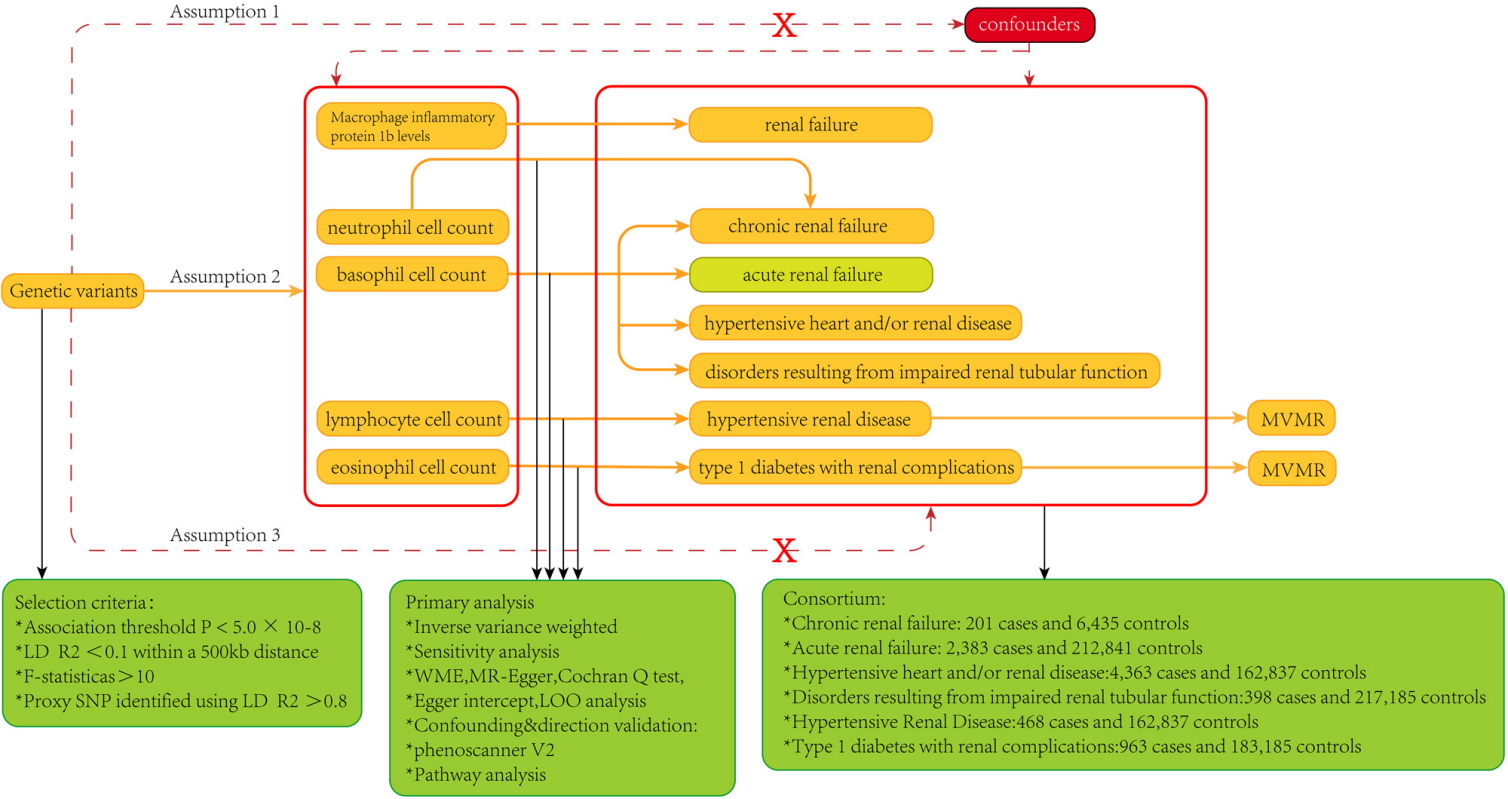
Study design and workflow of MR study.

This study used five cell traits among immune cells (neutrophils, basophils, lymphocytes, eosinophils, and macrophages) as exposure variables Figure 1. The outcome variables were various renal diseases (including chronic renal failure, acute renal failure, hypertensive heart or/and kidney disease, hypertensive renal disease, disorders resulting from impaired renal tubular function, type 1 diabetes with renal complications, and renal failure). The covariates used in multivariable MR are also four cell traits related to blood cells (neutrophil, basophil, lymphocyte, and eosinophil cell counts). All the above data are from the website (https://gwas.mrcieu.ac.uk/datasets). The above GWAS data are from the European population, and their brief information is shown in Tables 1 and 2.

**Table 1:** Brief description of the GWAS used in this study (exposure)

GWAS ID	Year	Trait	Consortium	Sample size	Number of SNPs
ieu-b-29	2020	Basophil cell count	Blood cell consortium	563,946	—
ieu-b-34	2020	Neutrophil cell count	Blood cell consortium	563,946	—
ieu-b-32	2020	Lymphocyte cell count	Blood cell consortium	563,946	—
ieu-b-33	2020	Eosinophil cell count	Blood cell consortium	563,946	—
ebi-a-GCST004433	2016	Macrophage inflammatory protein 1b levels	NA	8,243	9,802,973

**Table 2:** Brief description of the GWAS data used in this study (outcome)

GWAS ID	Year	Trait	Consortium	Sample size	Number of SNPs
ukb-e-N18_AFR	2020	Chronic renal failure	NA	6,636	15,240,712
finn-b-N14_ACUTERENFAIL	2021	Acute renal failure	NA	—	16,380,456
finn-b-I9_HYPTENSHR	2021	Hypertensive heart and/or renal disease	NA	—	16,380,199
finn-b-I9_HYPTENSRD	2021	Hypertensive renal disease	NA	—	16,380,163
finn-b-N14_DISIMPAIRRENTUB	2021	Disorders resulting from impaired renal tubular function	NA	—	16,380,463
finn-b-E4_DM1REN	2021	Type 1 diabetes with renal complications	NA	—	16,380,334
finn-b-N14_RENFAIL	2021	Renal failure	NA	—	16,380,466

### 2.2 Data sorting

**Figure 2:**
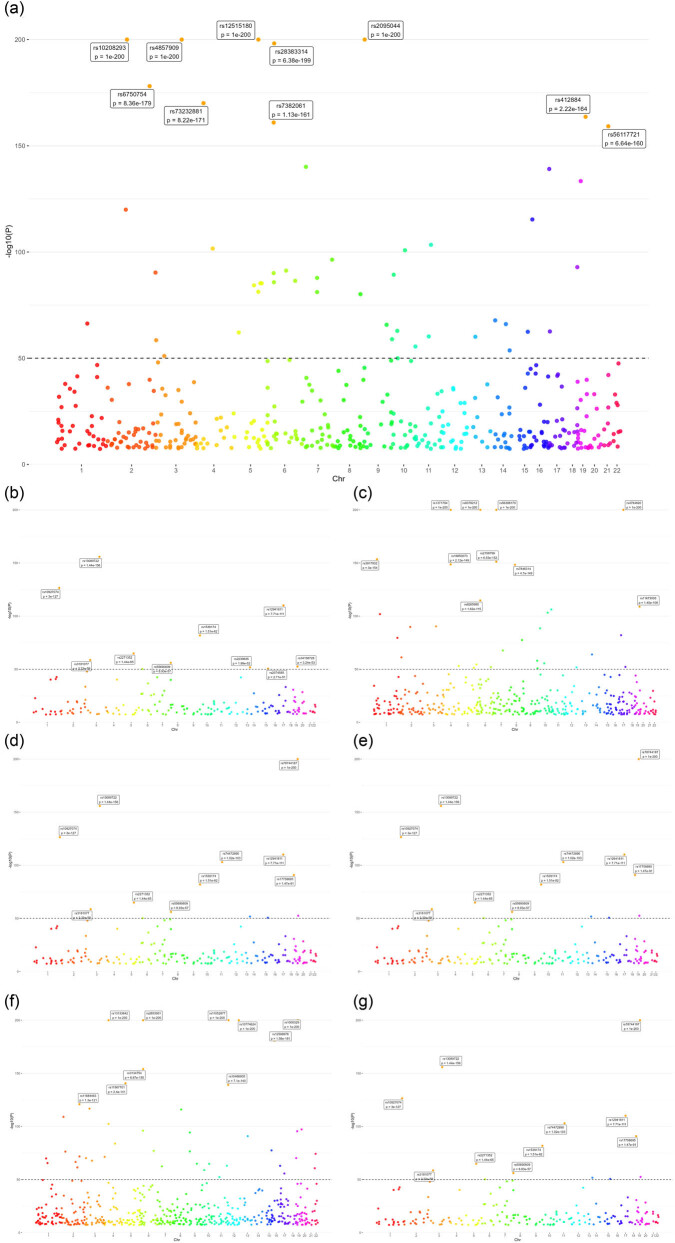
Manhattan map of chromosome locus in SNPs with different phenotypes. (a) Eosinophil cell count on type 1 diabetes with renal complications, (b) basophil cell count on chronic renal failure, (c) neutrophil cell count on chronic renal failure, (d) basophil cell count on acute renal failure, (e) basophils cell count on hypertensive heart and/or renal disease, (f) lymphocyte cell count on hypertensive renal disease, and (g) basophils cell count on disorders resulting from impaired renal tubular function.

To avoid bias caused by strong linkage disequilibrium (LD) between single nucleotide polymorphisms (SNPs) in MR analysis, SNPs that are independent of each other and have genome-wide significance in association with four different cell types are selected from the database as IVs. The screening criteria are: (1) With the whole gene information of the European 1000 Genome Project as a reference, four cell traits have genome-wide significance (*P* < 5 × 10^−8^); (2) Physical distance between two genes >10,000 kb; (3) The *R* ^2^ threshold of LD between genes was <0.01 [17]. Table 3 shows the basic information about some SNPs associated with basophil cell count. From Figure 2, we can see the chr sites in SNPs of different phenotypes).

**Table 3:** Basic information on some SNPs associated with basophil cell count

SNP	EA	OA	Beta	eaf	chr	pos	se	*p* val
rs10006833	C	T	−0.01561	0.209061	4	9,953,097	0.002521	6.22 × 10^−10^
rs10734121	A	G	−0.02543	0.845295	11	89,656,239	0.002837	3.28 × 10^−19^
rs10746147	G	A	−0.0224	0.926163	12	80,316,758	0.003927	1.20 × 10^−8^
rs10835333	G	A	0.014555	0.349229	11	3,957,766	0.002161	1.68 × 10^−11^
rs10844657	T	C	0.012373	0.339731	12	9,893,213	0.002168	1.18 × 10^−8^
rs1086893	C	T	0.029865	0.343174	1	2.13 × 10^8^	0.002162	2.41 × 10^−43^
rs10883359	G	A	−0.01313	0.284616	10	1.01 × 10^8^	0.002268	7.30 × 10^−9^
rs10906375	G	A	−0.01249	0.300993	10	13,498,371	0.002234	2.29 × 10^−8^
rs10927074	C	T	0.079216	0.892265	1	2.36 × 10^8^	0.0033	3.00 × 10^−127^
rs11097787	T	C	−0.0145	0.402632	4	1.03 × 10^8^	0.002087	3.83 × 10^−12^
rs7819602	G	C	0.016196	0.612901	8	10,726,842	0.002115	1.98 × 10^−14^
rs7832357	G	A	−0.01543	0.342269	8	1.27 × 10^8^	0.002158	8.90 × 10^−13^
rs79140637	A	G	−0.02748	0.054158	2	65,084,123	0.004583	2.09 × 10^−9^
rs8113682	G	T	−0.02161	0.747423	19	19,743,730	0.002364	6.41 × 10^−20^
rs875740	A	C	−0.01794	0.665329	16	16,123,048	0.002172	1.52 × 10^−16^
rs905670	A	G	−0.01431	0.350304	6	90,958,502	0.002142	2.49 × 10^−11^
rs915125	T	C	−0.02925	0.281093	6	82,463,376	0.002288	2.18 × 10^−37^
rs9274351	A	T	0.019736	0.196595	6	32,632,425	0.002822	2.79 × 10^−12^
rs9376098	A	T	0.023167	0.349186	6	1.35 × 10^8^	0.002147	4.15 × 10^−27^
rs9928015	T	G	−0.01499	0.302009	16	57,570,561	0.002233	2.01 × 10^−11^

### 2.3 Statistical treatment

#### 2.3.1 Principal analysis method

##### 2.3.1.1 Inverse variance weighted (IVW)

The inverse variance weighting method is the standard method for MR data aggregation [18]. It does not require individual-level data, and can directly use summary data to calculate the causal effect value. In the data using multiple genetic variations as IVs, for the *j*th IV, if the relevant assumptions of IVs are met, the relevant assumptions of IVs are satisfied, the estimated value \[{\hat{\beta }}_{j}]\] of the causal effect of exposure on the results is the ratio of the estimated value \[{\hat{\Gamma }}_{j}]\] of the correlation between the \[j]\] th genetic variation and the results and its estimated value \[{\hat{\gamma }}_{j}]\] of the correlation with exposure [19], namely,

\[{\hat{\beta }}_{j}=\frac{{\hat{\Gamma }}_{j}}{{\hat{\gamma }}_{j}}.]\]

If the genetic variation is uncorrelated (non-LD), the estimated values corresponding to each genetic variation can be summed into a weighted estimate of the whole, namely,

\[{\hat{\beta }}_{\text{IVW}}=\frac{{\sum }_{j}{w}_{j}{\hat{\beta }}_{j}}{{\sum }_{j}{w}_{j}},{w}_{j}=\frac{{\hat{\gamma }}_{j}^{2}}{{{\sigma }_{{Y}_{j}}}^{2}},]\]

where \[{{\sigma }_{{Y}_{j}}}^{2}]\] is the variance of the estimated gene result association of the \[j]\] th IV. If there is no correlation between genetic variables, the estimated value of IVW is equal to the estimated value of two-stage least square method for individual-level data [20]. However, like all IV methods, IVW methods are also vulnerable to weak instrumental bias. A simulation study shows that the weak instrumental bias level of IVW method is the same as that of two-stage least square method, and its size can be quantified by \[F]\] statistics [21].

#### 2.3.2 MR-Egger regression

MR-Egger regression is a method proposed in recent years to detect and adjust the pleiotropy of MR analysis. In this method, given a group of genetic variations, first, the correlation \[{\hat{\Gamma }}_{j}]\] between each genetic variation and the results and the correlation \[{\hat{\gamma }}_{j}]\] between genetic variation and exposure are estimated, and then the linear function is fitted [22].

\[{\hat{\Gamma }}_{j}={\beta }_{0\text{E}}+{\beta }_{\text{E}}{\hat{\gamma }}_{j}.]\]

The estimated value \[{\hat{\beta }}_{\text{E}}]\] of the causal effect of exposure on the results can be calculated using the following formula:

\[{\hat{\beta }}_{\text{E}}=\frac{\mathrm{cov}(\hat{\Gamma },\hat{\gamma })}{\mathrm{var}(\hat{\gamma })}.]\]

The intercept estimate \[{\hat{\beta }}_{0\text{E}}]\] of MR-Egger regression is the average of the estimates of the multiple effects of each genetic variation. MR-Egger method relaxed the requirement that there are no multiple validities between genetic variations in IVW method. It assumes that the instrumental exposure and instrumental outcome associations are independent. This is called the instrument strength dependent on direct effect (InSIDE) hypothesis [23], which is relatively weak compared with the strict exclusion limit standard. However, both IVW and MR-Egger regression methods theoretically need to assume that the gene variation exposure association is a measurement error (NOME) [24]. The cost of MR-Egger’s relaxation of the multiple validity hypothesis is that it violates the NOME hypothesis, resulting in greater bias than the IVW estimate, and is particularly vulnerable to the impact of weak instrumental bias [25]. In addition, MR Egger regression can detect pleiotropy only when gene pleiotropy has directionality (that is, pleiotropy has a non-zero mean value) [26]. Because only in this case can \[{\beta }_{0\text{E}}]\] be a non-zero value. For example, when all genetic variations show pleiotropy but their directions are different, and they offset each other at the average level (this situation is called balanced pleiotropy [27]), MR-Egger regression cannot detect pleiotropy.

#### 2.3.3 Median estimation

Median estimation includes simple median estimation, weighted median estimation, and penalty-weighted median estimation. Simple median estimation methods are very easy to understand. Let \[{\hat{\beta }}_{j}]\] represent the estimated value of the *exposure–outcome* effect corresponding to the \[j]\] th genetic variation (from the smallest to the largest). If the total number of genetic variations is an odd number \[(J=2k+1)]\] , the simple median estimate will take its median value \[{\hat{\beta }}_{k+1}]\] . If it is an even number \[(J=2k)]\] , its estimated value is \[\frac{1}{2}({\hat{\beta }}_{k}+{\hat{\beta }}_{k+1})]\] . We can understand a simple median estimation as a weighted median estimation with the same weight. However, this method is inefficient when the estimation accuracy corresponding to different genetic variations varies greatly [28]. The weighted median estimation considers the problem of the large difference in estimation accuracy. In this method, let \[{w}_{j}]\] be the weight of the \[j]\] th genetic variation estimate, and let \[{s}_{j}={\sum }_{k=1}^{J}{w}_{k}]\] be the sum of the weights of \[j]\] estimates (arranged from the smallest to the largest). If normalized, \[{s}_{j}]\] equals 1, the weighted median estimate is the estimate that \[{p}_{j}]\] equals 50%, here \[{p}_{j}=100\left(\phantom{\rule[-0.75em]{}{0ex}},{s}_{j}-\frac{{w}_{j}}{2}\right)]\] . Similar to the IVW method, the weight \[{w}_{j}]\] of this method generally uses the inverse weight of variance of each genetic variation [29]:

\[{w}_{j}=\frac{{\hat{\gamma }}_{j}^{2}}{{\sigma }_{{Y}_{j}}^{2}}.]\]

It is worth noting that the simple median estimation requires that at least 50% of the genetic variation is an effective IV, while the weighted median estimation only requires that at least 50% of the weight contributed by the genetic variation is effective. Although the existence of an invalid IV does not affect the asymptotic unbiasedness of the median estimate, it may cause bias in a limited sample. When the estimation of invalid IV is unbalanced on both sides of the real causal effect (for example, there are multiple invalid IVs in a study, and the estimated values of these IVs are all greater or less than the real estimated values), bias may occur. In this case, a penalty-weighted median estimation can be used for correction to reduce the weight of genetic variation with heterogeneous estimates. When using this method, first the heterogeneity between estimates is quantified with Cochran’s *Q* value [30]:

\[Q=\sum _{j}{Q}_{j}=\sum _{j}{w}_{j}{({\hat{\beta }}_{j}-\hat{\beta })}^{2},]\]

where \[\hat{\beta }]\] is the estimated value obtained by the IVW method. Under the null hypothesis that all genetic variations are valid IV and all variables can identify the same causal relationship, \[{Q}_{j}]\] follows the chi-square distribution with a degree of freedom of 1. Through this distribution, the *P* value (expressed in \[{q}_{j}]\] ) corresponding to the \[{Q}_{j}]\] value of each genetic variation is determined. Then, the weight is multiplied by the *P* value and then by 20 (if the *P* value is greater than 0.05, multiply by 1) to punish. Finally, the weight \[({w}_{j}^{\ast })]\] after collection and punishment are obtained [31]:

\[{w}_{j}^{\ast }={w}_{j}\times \hspace{.25em}\min (1,20{q}_{j}).]\]

#### 2.3.4 Multivariate MR analysis

A multivariate MR method was used to correct covariates: neutrophil, basophil, lymphocyte, and eosinophil cell counts, and test whether they have an impact on the association between exposure and different kidney diseases. SNPs used as IVs in multivariate MR should meet the following conditions: (1) SNPs are associated with all exposure factors in the model; (2) the outcome variables will not be affected by other ways; and (3) the number of SNPs is greater than the number of exposure factors [32].

The above analysis was completed with R4.1.2 software. MR-IVW, MR-WME, and MR-Egger are completed with R software package TwoSampleMR, and MR-PRESSO and multivariate MR are completed with R software packages MR-PRESSO and MR, respectively. The evaluation indexes were odds ratio (OR) and 95% confidence interval (95% CI). *P* ＜ 0.05 means the difference is statistically significant (bilateral) [33].

#### 2.3.5 Cross-traits LD score regression

LD refers to the phenomenon in a population where the frequency of simultaneous inheritance of two genes at different loci is significantly higher than the expected random frequency. The LD score reflects whether the biologically related variant genes of two phenotypes are in a high linkage imbalance state. LD score regression (LDSC) is essentially a linear regression, with input data being GWAS aggregated data. The *Z*-statistic of the genetic association between each variant and phenotype 1 is multiplied by the *Z*-statistic of the genetic association with phenotype 2, and the product of this statistic is then regressed with the LD score. The slope (coefficient) represents the genetic correlation. This study evaluated the genetic correlation between each phenotype using the recommended settings in the software package LDSC (v1.0.1). This software package has been widely used in most studies to identify genetic correlations between complex phenotypes and diseases. This study used European lineage information from the 1000 Genome Project as a reference for linkage imbalance analysis, consistent with the European ethnic origin of the GWAS sample.

## 3 Results

### 3.1 Two samples Mendelian randomized study results

#### 3.1.1 Determination of MR IVs

2391 SNPs that can effectively predict four different types of leukocyte cells in the leukocyte family at the genome level can be used as potential IVs. Considering that some SNPs are in LD, MRBase is further used (https://mrcieu.github.io/TwoSampleMR/) to estimate the LD between SNPs (LD *R* ^2^ < 0.05). After the “LD lumped” step, 163 independent SNPs that are not in linkage imbalance are excluded. Finally, 2,187 SNPs were selected as IVs for subsequent MR analysis. The *F*-value is used to define “weak IV”, so the bias of IV is minimized.

#### 3.1.2 MR analysis results

##### 3.1.2.1 Increased neutrophil cell count on chronic renal failure

IVW analysis results showed that increased neutrophil cell count was a risk factor for chronic renal failure (OR = 2.0245861, 95% CI = 1.1231207–3.649606, *P* = 0.01896524), and similar results were obtained in weighted median (OR = 2.7653808, 95% CI = 1.0357616–7.383293, *P* = 0.04234353). MR-Egger regression results showed that the intercept was 0.019347238 (close to 0), *P* = 0.19151, indicating that genetic pleiotropy would not bias the results of this study.

##### 3.1.2.2 Increased basophil cell count on chronic renal failure

Mendelian randomized study of two samples showed that increased basophil cell count was a risk factor for chronic renal failure (OR = 3.975935, 95% CI = 1.4871198–10.62998, *P* = 0.005942755). MR-Egger regression results showed that the intercept was 0.028891206 (close to 0), *P* = 0.247636, indicating that genetic multiplicity would not bias the results of this study.

##### 3.1.2.3 Increased basophil cell count on acute renal failure

An MR study showed that increased basophil cell count was not a risk factor for acute renal failure. IVW results showed that (OR = 1.160434, 95% CI = 0.9455132–1.424207, *P* = 0.15448828), while similar results were obtained in weighted median (OR = 1.413948, 95% CI = 0.9786767–2.04288, *P* = 0.06501094), and MR-Egger (OR = 1.423131, 95% CI = 0.977184–2.072588, *P* = 0.06738522). MR-Egger regression results showed that the intercept was –0.00608 (close to 0), *P* = 0.206087, indicating that genetic pleiotropy would not bias the results of this study.

##### 3.1.2.4 Increased basophil cell count on hypertensive heart and/or renal disease

IVW analysis results showed that increased basophil cell count was a protective factor for hypertensive heart and/or renal disease (OR = 0.7716065, 95% CI = 0.6484979–0.9180856, *P* = 0.003458707). MR-Egger regression results showed that the intercept was –0.00248 (close to 0), *P* = 0.543814, indicating that genetic pleiotropy would not bias the results of this study.

##### 3.1.2.5 Increased basophil cell count on disorders resulting from impaired renal tubular function

IVW analysis results showed that increased basophil cell count was a risk factor for disorders resulting from impaired renal tubular function (OR = 1.648131, 95% CI = 1.010116–2.689133, *P* = 0.04546835), and similar results were obtained in weighted median (OR = 2.648595, 95% CI = 1.144769–6.127922, *P* = 0.02285107). MR-Egger regression results showed that the intercept was –0.01197 (close to 0), *P* = 0.296908, indicating that genetic pleiotropy would not bias the results of this study.

##### 3.1.2.6 Increased lymphocyte cell count on hypertensive renal disease

IVW analysis results showed that increased lymphocyte cell count was a risk factor for hypertensive renal disease (OR = 1.372961, 95% CI = 1.0189772–1.849915, *P* = 0.03719874). MR-Egger regression results showed that the intercept was –0.00548 (close to 0), *P* = 0.469176, indicating that genetic pleiotropy would not bias the results of this study.

##### 3.1.2.7 Increased eosinophil cell count on type 1 diabetes with renal complications

**Figure 3:**
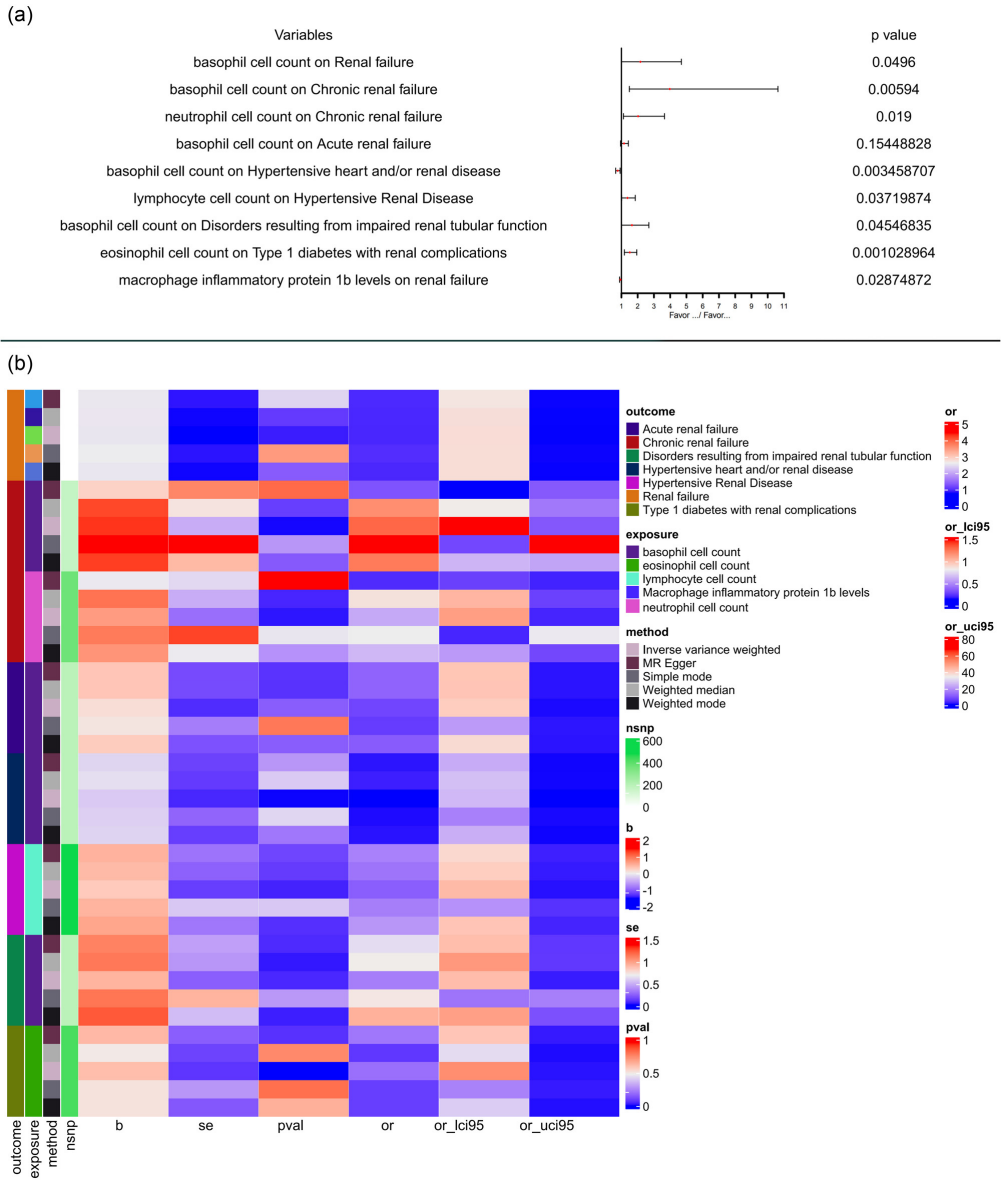
(a) The IVW method was used to detect the effects of different immune cell counts on different kidney diseases; (b) MR analysis tested the effects of different immune cell counts on seven types of kidney disease. The results obtained using five MR methods (IVW, MR-Egger, weighted median, simple mode, and weighted mode) are presented in the form of heatmap, representing causal estimation.

IVW analysis results showed that increased eosinophil cell count was a risk factor for type 1 diabetes with renal complications (OR = 1.516454, 95% CI = 1.1826453–1.944482, *P* = 0.001028964). MR-Egger regression results showed that the intercept was −0.00148 (close to 0), *P* = 0.827094, indicating that genetic pleiotropy would not bias the results of this study, Figure 3.

##### 3.1.2.8 Macrophage inflammatory protein 1b levels on Renal failure

IVW analysis results showed that Macrophage inflammatory protein 1b levels was a protective factor for renal failure (OR = 0.9381862, 95% CI = 0.8860402–0.9934013, *P* = 0.02874872), while the MR-Egger regression results showed the intercept was −0.007370909 (close to 0), *P* = 0.7695226, indicating that genetic pleiotropy would not bias the results of this study.

#### 3.1.3 Sensitivity analysis

**Figure 4:**
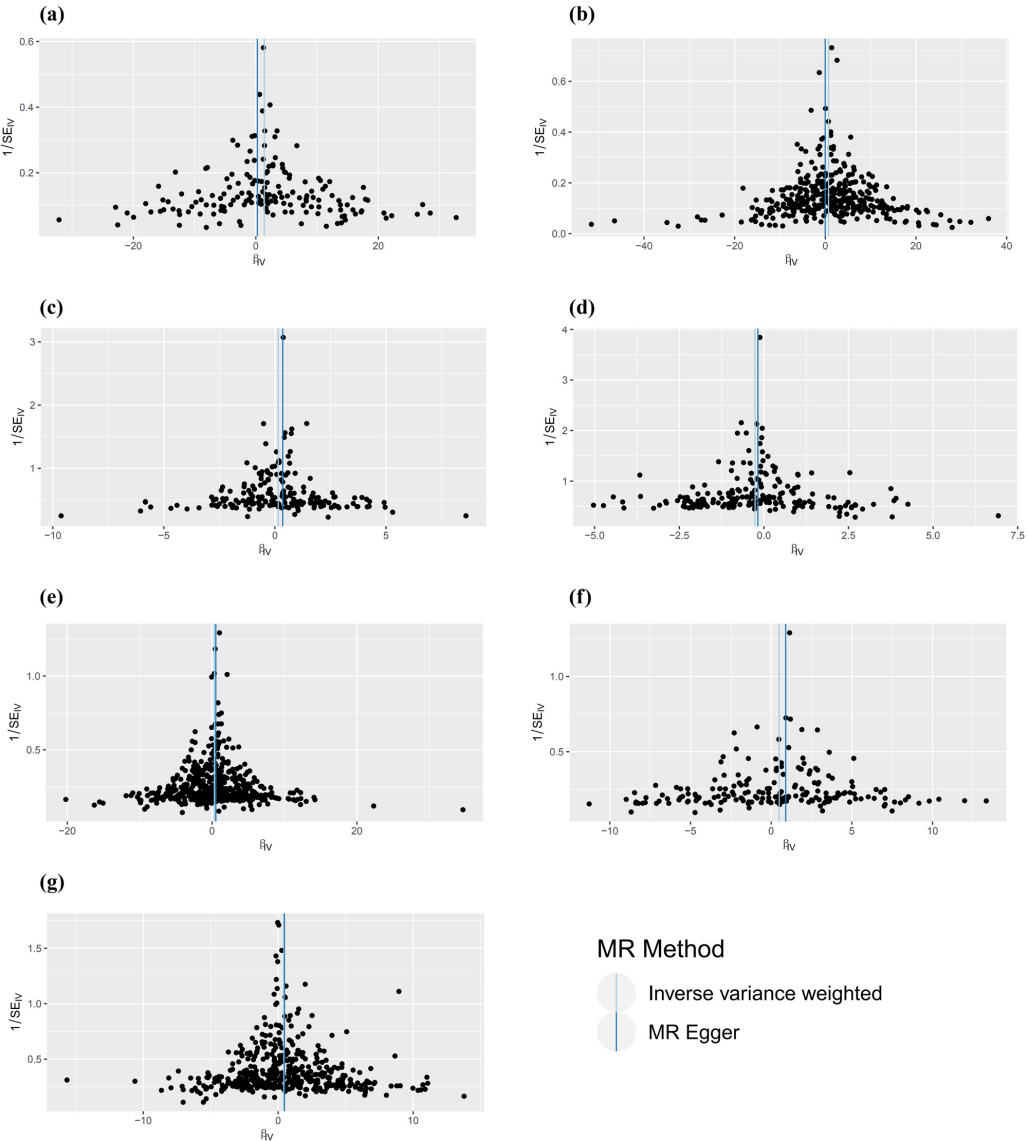
MR analysis tested the influence of four kinds of cell counts in the leukocyte family on nephropathy. The results of IVW and MR-Egger are presented in the form of a funnel plot. (a) Basophil cell count on chronic renal failure, (b) neutrophil cell count on chronic renal failure, (c) basophil cell count on acute renal failure, (d) basophil cell count on hypertensive heart and/or renal disease, (e) lymphocyte cell count on hypertensive renal disease, (f) basophils cell count on disorders resulting from impaired renal tubular function, and (g) eosinophil cell count on type 1 diabetes with renal complications.

Cochran Q test of IVW and MR-Egger regression of eight groups of two samples showed that there was no heterogeneity in SNPs. There was no significant statistical difference between Egger-intercept and 0 (*P* > 0.05), so we can think that there is no level pleiotropy of SNPs. Funnel diagram shows that when a single SNP is used as IVs, the points representing the causal correlation effect are symmetrically distributed, indicating that the causal correlation is less likely to be affected by potential bias, Figure 4. The results of the “Leave one out” sensitivity analysis show that the IVW analysis results of the remaining SNPs are similar to those of all SNPs after the SNPs are eliminated in turn. No SNP that has a greater impact on the causal association estimate is found [34].

### 3.2 Multivariate MR study results

#### 3.2.1 Increased eosinophil cell count on type 1 diabetes with renal complications

After multivariable MR was used to correct covariates (increased neutrophil, basophil, and lymphocyte cell counts), the correlation effect between eosinophil cell count and type 1 diabetes with renal complications was still statistically significant (*P* = 0.02201152).

#### 3.2.2 Increased lymphocyte cell count on hypertensive renal disease

**Figure 5:**
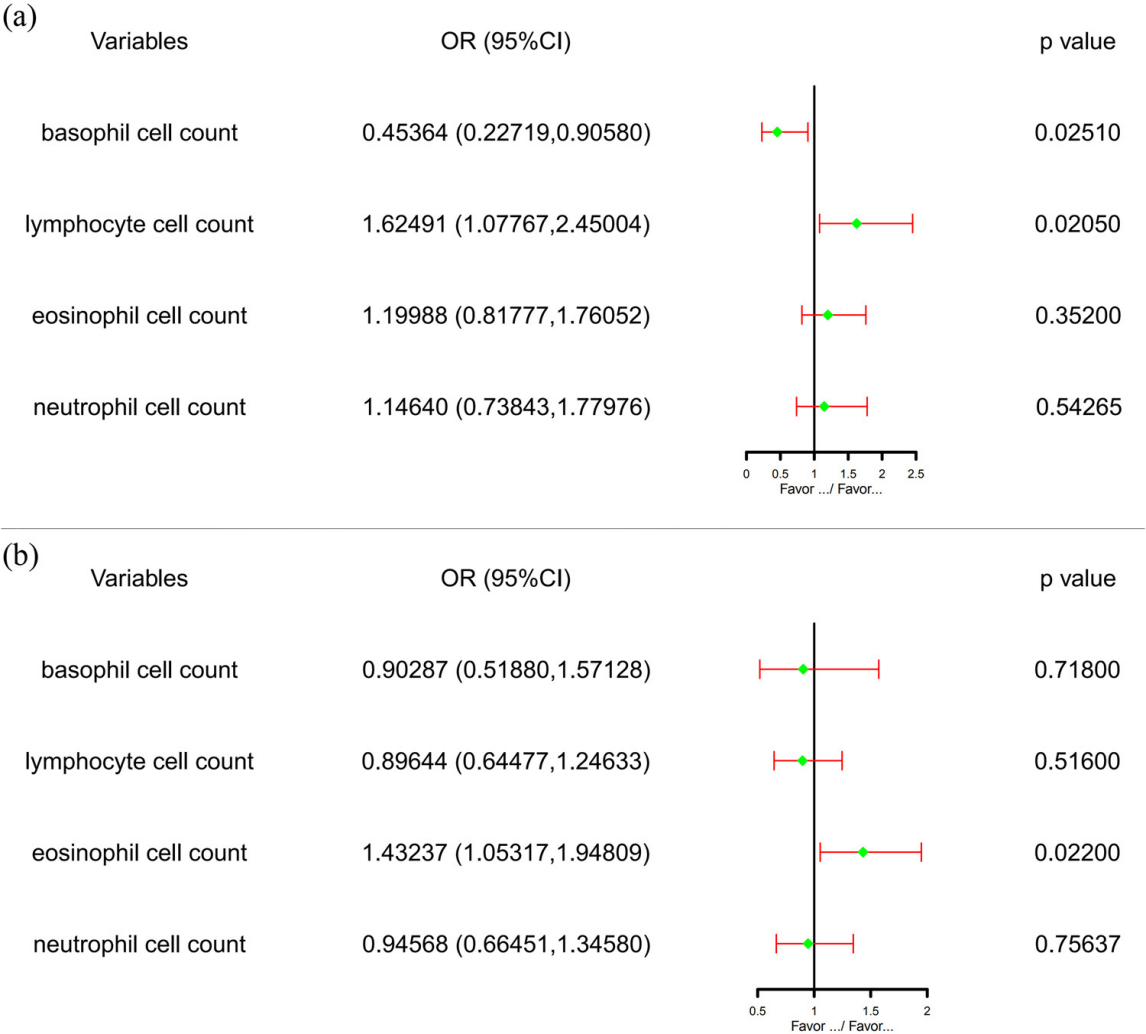
MVMR results. (a) The effect of lymphocyte cell count on hypertensive renal disease after correcting basophil, eosinophil, and neutrophil cell counts and (b) the effect of eosinophil cell count on type 1 diabetes with renal complications after correcting basophil, lymphocyte, and neutrophil cell counts.

After adjusting covariates (neutrophil, basophil, and eosinophil cell counts) with multivariable MR (MVMR), the correlation effect between lymphocyte cell count and hypertensive renal disease was still statistically significant (*P* = 0.02050226). It is worth noting that in the process of MVMR, the correlation effect between increased basophil cell count and hypertensive renal disease was also statistically significant (*P* = 0.02506460), Figure 5.

### 3.3 Genetic correlation test between immune cells and different kidney diseases

Regression analysis of LD score (LDSC) between exposure and outcome revealed that kidney diseases with negative genetic correlation with basophil cell count were chronic renal failure (rg = NA, *P* = NA), acute renal failure (rg = NA, *P* = NA), and disorders resulting from impaired renal tubular function (rg = NA, *P* = NA). The kidney disease with negative genetic correlation with neutrophil cell count is chronic renal failure (rg = NA, *P* = NA). Macrophage inflammatory protein 1b levels have no genetic correlation with renal failure (rg = −0.123461, *P* = 0.814559). There is no genetic correlation between basophil cell count and hypertensive heart and/or renal disease (rg = −0.001703, *P* = 0.973137). There is a genetic correlation between lymphocyte cell count and hypertensive renal disease, but the correlation is extremely low (rg = 0.199335, *P* = 0.031153). There is a genetic correlation between eosinophil cell count and Type 1 diabetes with renal complications, but the correlation is extremely low (rg = 0.175011, *P* = 0.048723).

## 4 Discussion

For the first time, Mendelian randomized analysis was used in our study to explore the potential causal relationship between four types of cells count (basophils, eosinophils, neutrophils, and lymphocytes) in the white blood cell family and different kidney diseases (chronic renal failure, acute renal failure, hypertensive heart and/or renal disease, disorders resulting from impaired renal tubular function, hypertensive renal disease, and type 1 diabetes with renal complications). We found that increased basophil cell count will increase the relative risk of chronic renal failure and disorders resulting from impaired renal tubular function, and reduce the risk of hypertensive heart and/or renal disease, while increased basophil cell count will not increase the relative risk of acute renal failure, so we speculate that increased basophil cell count affecting renal failure is a relatively long process. In addition, increased neutrophil cell count can increase the risk of chronic renal failure, increased lymphocyte cell count can increase the relative risk of hypertensive renal disease, increased eosinophil cell count can increase the relative risk of type 1 diabetes with renal complications, and macrophage inflammatory protein 1b levels are protective factors for renal failure. The above relationships have been confirmed in the weighted median method, MR-Egger regression, WME, and MR-presso. Second, MR-Egger intercept detection and test showed that the genetic variables included above did not have any pleiotropy. These results indicate that an increase in the four cell counts in the cell family may serve as a risk factor to promote the occurrence of a certain type of kidney disease, and macrophage inflammatory protein 1b levels can serve as protective factors to reduce the occurrence of renal failure. It is worth mentioning that this study also carried out MVMR for increased lymphocyte cell count on the occurrence of hypertensive renal disease and increased eosinophil cell count in type 1 diabetes with renal complications. The results show that after correcting the covariates, the results of increased lymphocyte cell count on hypertensive renal disease and increased eosinophil cell count on type 1 diabetes with renal complications are still significant.

**Figure 6:**
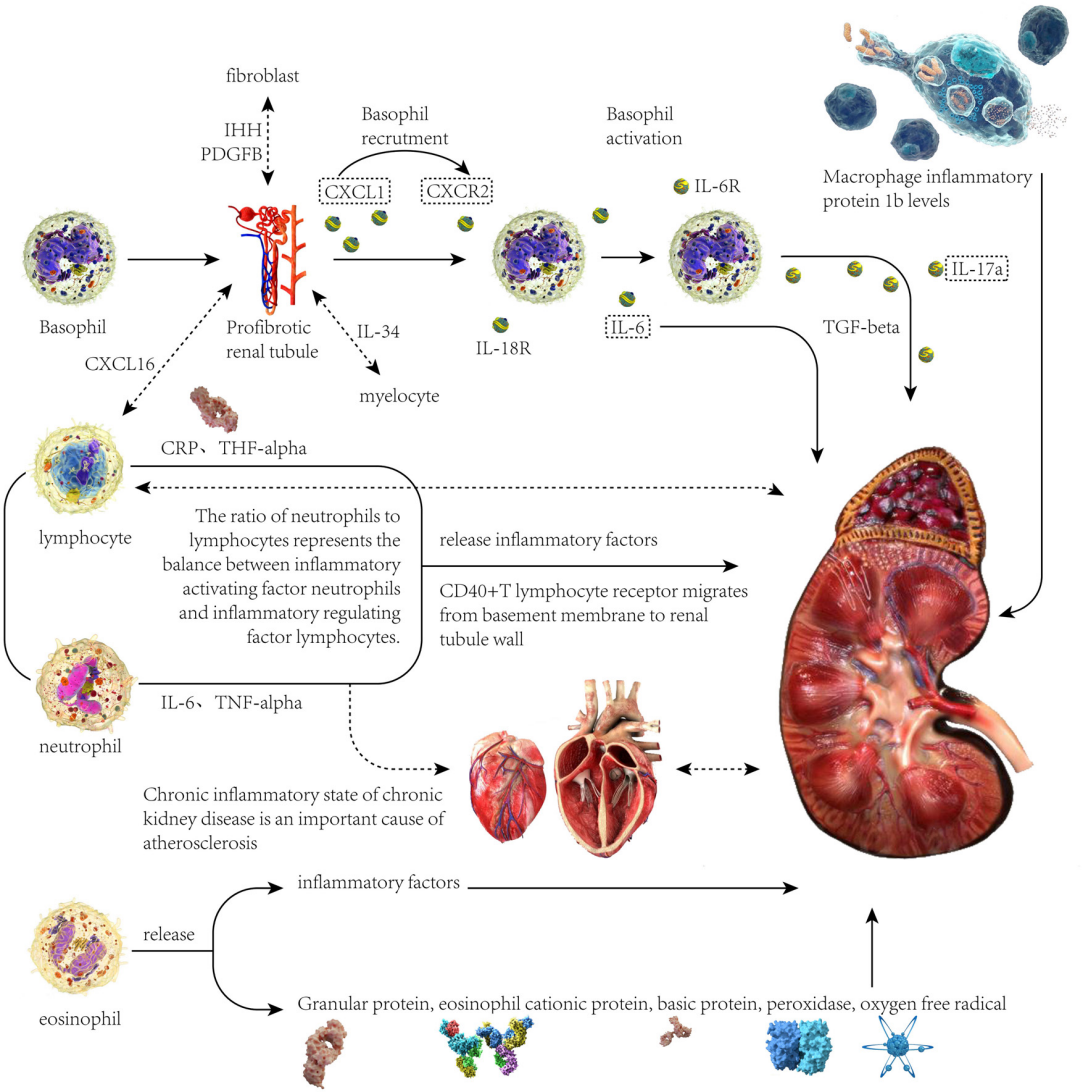
Abstract figure, the pathological mechanism of immune cells and kidney disease.

The exact mechanism by which different immune cell counts increase and lead to different kidney diseases is currently unclear, but some studies speculate that this may be the result of multiple factors working together. Therefore, we will elaborate on each one, Figure 6.

### 4.1 Causal relationship between increased basophil count and renal diseases

Renal fibrosis is often referred to as unresolved inflammation [35]. Studies have shown that basophils play an active role in renal fibrosis. There is also increasing evidence that proximal tubular (PT) cells play an important role in the development of renal diseases and renal fibrosis [36]. Genetic studies have shown that PT cells are rich in genes that can lead to renal dysfunction [37]. “Fibrosis PT” cells interact with resident fibroblasts through intermittent hypercapnic hypoxia and platelet-derived growth factor B, with myeloid cells through IL-34, and with lymphocytes through C-X-C motif chemokine 16 (CXCL16). Previous studies [38] established the key role of IL-34 and CXCL16 in renal fibrosis. Fibrogenic PT cells expressing CXCL1 may be responsible for basophil recruitment, although the role of other pathways remains to be determined. A previous paper pointed out the role of CXCL1 in the recruitment of granulocytes, including basophils [39]. CXCL1 expression was also enriched in tubular cells with weak expression of proximal tubular marker HNF4A and expression of Henle down loop marker, indicating that similar altered cell populations may extend to the margin of PT.

Some studies have emphasized the increase in the number of basophils in renal fibrosis. It is suggested that the increased matrix expression of IL-18 and IL-33 may play a role in activating basophils and stimulating the secretion of IL-6. Previous studies have shown that blocking IL-18 and IL-33 can improve renal fibrosis [40]. IL-6 has a pleiotropic effect on inflammation and immune response, and it may have related proinflammatory or anti-inflammatory properties [41]. In animal studies, mice treated with IL-6R antibodies were partially immune to renal fibrosis. Previous studies also showed that IL-6 promotes fibrosis in myocardial [42], pulmonary [43], and peritoneal [44] fibrosis, suggesting that IL-6 may be a common mediator of fibrosis. However, previous studies have failed to determine the source of IL-6 in fibrosis. Coppock and other scholars clarified that basophils are the main source of IL-6 in renal fibrosis. TH17 cells that respond to IL-6 may be downstream mediators of basophils in renal fibrosis. The research team has previously clarified the contribution of TH17 cells to renal fibrosis [45], and their role in pulmonary fibrosis has also been confirmed. TH17 cells can interact with fibroblasts to coordinate fiber inflammation [46]. In our study, we concluded that basophils have a positive causal relationship with nephropathy caused by chronic renal failure and renal tubular function damage.

### 4.2 Effect of increased eosinophils count on diabetes nephropathy

Some cytokines (such as IL-3, IL-5, GM-CSF, etc.) act on eosinophil cell lines, accelerating the proliferation and differentiation of bone marrow eosinophil hematopoietic progenitor cells. Activation increases eosinophil function and mobilize eosinophils to migrate locally [47]. It can involve pancreatic islet function, heart, skin, nerve, respiration, digestion, and other systems. The mechanism may be that eosinophils increase, infiltrate tissues extensively, and release a large number of cytotoxics and a variety of factors that cause inflammation and fibrosis, thus causing tissue damage [48]. It may be related to the infiltration of eosinophils and the release of inflammatory mediators. Eosinophils aggregate in renal tissue and release granular proteins after activation, including eosinophil cationic proteins, major basic proteins, peroxidase, oxygen free radicals, and other cytotoxic factors [49], thus causing renal damage. In our study, we concluded that eosinophils are a risk factor for diabetic nephropathy.

### 4.3 Effect of increased neutrophils count on chronic renal failure

The imbalance of the immune state characterized by a systemic inflammatory reaction and immune deficiency exists universally in patients with chronic renal failure. Inflammatory reaction related to ESRD is related to the activation of the natural immune system, mainly including the activation of neutrophils, macrophages, and monocytes, the expression of Toll-like receptors, the production and release of cytokines, and reactive oxygen-free radicals. At the same time, systemic inflammation will bring atherosclerosis, anemia, cachexia, and other complications [50]. Immune deficiency will lead to an increase in the incidence rate, severity, and mortality of various microbial infections, and become the second leading cause of death in patients with ESRD [51]. This is due to the weakened functions of neutrophils, monocytes, and macrophages in uremic patients with immune deficiency, the insufficient role of antigen-presenting cells in presenting defensive antigens, the reduced number of B lymphocytes producing antibodies, and the reduced function of the overall cellular immune function [52,53,54]. The study found that neutrophils in patients with chronic renal failure were significantly higher than those in healthy people, and were associated with IL-6 and TNF-α. The level was positively correlated. Turkmen et al. [55] found that neutrophils and TNF-α in patients with ESRD (including hemodialysis and peritoneal dialysis) present positive correlation. Domestic retrospective studies on patients with chronic kidney disease suggest that neutrophils in hemodialysis patients are positively correlated with hsCRP [56].

In most chronic kidney diseases, especially in patients with chronic renal failure, proteinuria is one of the common symptoms. On the one hand, the appearance of proteinuria indicates the damage to the glomerular filtration barrier, and on the other hand, the protein in urine will have an endogenous toxic effect on the kidney, which is closely related to the degree of renal fibrosis and glomerulosclerosis [57]. Binnetoğlu et al. [58] observed 1,000 patients with chronic kidney disease and found that neutrophils were strongly positively correlated with the occurrence and severity of 24h proteinuria. Studies have shown that the degree of albuminuria is related to the infiltration of inflammatory cells in the renal interstitium. Early inflammation damages the glomerular capillary filtration barrier, which will lead to the emergence of albuminuria. Persistent inflammation and albuminuria cause CD40+T lymphocyte receptors to migrate from the basement membrane to the renal tubule wall. These receptors release inflammatory factors after binding with T lymphocytes, causing inflammation in the tubulointerstitium and subsequent renal function damage [59]. In this study, we concluded that neutrophils are a risk factor for chronic renal failure.

### 4.4 Relationship between lymphocyte ratio and renal diseases

In the pathogenesis of some kidney diseases, cellular immune disorder plays a major role, and cellular immunity is played by T cells through the release of cytokines. According to different immune effects, T cells can be roughly divided into three subsets: CD4+helper T cells (Th cells), CD8+cytotoxic T cells (Ts cells), and CD4+CD25+regulatory T cells (Treg cells). Most previous studies believed that the occurrence of T lymphocyte and kidney diseases was mainly manifested in the abnormal number and function of T lymphocyte subsets, which promoted the dysfunction of T cells and produced some pathogenic factors, thus leading to the occurrence of diseases. It has been reported in the literature [60] that high levels of cytokines released by T lymphocyte subsets are associated with persistent proteinuria in some patients with kidney diseases. The helper T cells carry CD4+surface antigen and can differentiate into helper T lymphocyte 1 (Th1), Th2, Th17, T follicular helper cells (TFH), and other cell subsets. The proportion of Th1, Th2, and Th17 cells is crucial for the monitoring of immunity [61]. TFH is a newly discovered cell subpopulation related to the pathogenesis of kidney disease in recent years. The combination of CD40 ligand (CD40L) on TFH with CD40 on the surface of B cells is the main mechanism for B cells to produce plasma cells, antibodies, and immunoglobulin class conversion. Some studies [62] found that CD40L in patients with kidney disease was significantly lower than that in normal people by measuring CD40L on TFH cells, indicating that the decrease in blood IgG level in patients with kidney disease was related to the weakening of CD40/CD40L response. The main effect of Th1 cells is to induce cellular immunity by releasing cytokines. The main effect of Th2 cells is to help B cells activate and produce antibodies. Th1 and Th2 maintain the balance of cellular immunity and humoral immunity of the body, respectively, which is in dynamic balance under normal conditions. It has been reported in foreign studies [63] that the imbalance of Th1/Th2/Th17 leads to an increase in the secretion of granulocyte-macrophage colony-stimulating factor (TNF) related activation-induced cytokine (TRANCE) of Th1 and Th17 cells, which further leads to foot cell damage and proteinuria. It indicates that the pathogenesis of kidney disease is related to the disorder of T cell immune function caused by Th1/Th2/Th17. Th17 is a newly discovered CD4+T cell subpopulation that mainly secretes the cytokine IL-17. Many kinds of literature reported that Th17 is involved in inflammatory reactions and autoimmune diseases. It has been pointed out in foreign literature [64] that Th17 and IL-17 may participate in the pathogenesis of kidney disease by reducing the expression of Podophysin protein in podocytes and inducing podocyte apoptosis. Some studies [65] show that IL-17 is highly expressed in the kidney. Nuclear factor kappa-B (NF-kB) induces podocyte apoptosis in a dependent manner, aggravates kidney damage, and thus leads to kidney disease. When some foreign scholars [66] studied the role of regulatory T cells (Treg cells) in the pathogenesis of renal diseases, they found that the number of Treg cells decreased in the onset period and increased in the remission period, which proved that Treg cells were involved in the induction and remission process of nephrotic syndrome. When some scholars studied the relationship between Treg cells and CD80 expression in renal biopsy tissues of PNS children, they found that the number of Treg cells in renal tissues with positive CD80 expression decreased, indicating that the decrease in the anti-inflammatory environment may be the reason for the increase in CD80 expression [67]. Both Th17 and Treg cells belong to CD4+T lymphocyte subsets, but they are antagonistic to each other in the process of exerting immune effects and jointly maintaining the balance of immune function. If the balance is broken, a series of immunopathological reactions will occur [68]. Th17/Treg balance is necessary in autoimmune diseases as Th17 cells promote autoimmune and inflammatory reactions, while Treg cells inhibit these phenomena and maintain immune homeostasis [69]. A study [70] found that the proportion of Th17 cells in patients with kidney disease before treatment was significantly higher than that in the healthy control group, while the proportion of Treg cells was significantly lower than that in the healthy control group, indicating that both types of cells were involved in the pathogenesis of kidney disease. The ratio of Th17/Treg cells significantly decreased after hormone treatment, which was reversed compared with that before treatment, indicating that Th17/Treg immune imbalance may be involved in the pathogenesis of kidney disease. In this study, we concluded that lymphocytes are a risk factor for hypertensive nephropathy.

### 4.5 Relationship between neutrophil/lymphocyte ratio and renal diseases

Cardiovascular events are the most common complication of patients with chronic renal failure and the most common cause of death, accounting for about 50% of all causes of death [71]. The concept of malnutrition inflammation atherosclerosis syndrome has been put forward by the academic community [72]. On this basis, foreign scholars selected 225 patients with stage 3–5 chronic kidney disease for a clinical cohort study. The results showed that there was a strong correlation between the neutrophil/lymphocyte ratio and the blood flow regulation and relaxation function of the blood vessels, suggesting that the neutrophil/lymphocyte ratio can more sensitively reflect the functional status of the vascular endothelium [73]. In another study, 56 patients with ESRD were observed. After comparing the ratio of neutrophils/lymphocytes with the degree of calcification of carotid and coronary arteries by ultrasound and CT, it was found that there was a linear positive correlation between the ratio of neutrophils/lymphocytes and the degree of calcification of coronary and carotid arteries. Neutrophils play an important role in early endothelial dysfunction and the initial stage of atherosclerotic plaque formation. After activation, neutrophils can adhere to and penetrate vascular endothelial cells, release certain reactive oxygen free radicals, cytokines, and hydrolases, increase the damage of vascular endothelium, and promote the initiating factors of atherosclerotic plaque formation. *In vivo* experiments have found that there is a correlation between the number of neutrophils in the circulatory system and atherosclerotic injury. Reducing the content of neutrophils can significantly alleviate the damage to vascular endothelium. The number of lymphocytes can also reflect the progression of atherosclerotic disease, because there is apoptosis of lymphocytes on the vascular endothelium damaged by atherosclerosis, and the number of lymphocytes can simply reflect the stress response caused by adrenocortical hormone, and then indirectly reflect the stress damage of vascular endothelium. Neutrophils and lymphocytes can reflect the oxidative stress state and cytokine release caused by sympathetic nerve activation, and then can sensitively reflect the inflammation and oxidative stress in the body, thus becoming an independent predictor of vascular endothelial function damage in patients with chronic renal failure, and can well predict the prognosis of patients after cardiovascular events [74]. In addition, the abnormal metabolism of trace elements and the deposition of calcium salt in the inner wall of blood vessels are the main causes of atherosclerosis. For patients with chronic kidney disease, especially end-stage kidney disease, atherosclerosis, inflammation, and vascular calcification are the main risk factors for cardiovascular events and even life-threatening emergencies, and the assessment of their aortic calcification is an important indicator for predicting future risk events. In a cross-sectional observation of 56 patients with ESRD, it was found that the ratio of neutrophils and lymphocytes was positively correlated with the degree of arterial calcification, so calculating the ratio of neutrophils and lymphocytes could better predict the degree of vascular calcification in ESRD patients, thus providing a basis for assessing their risk of cardiovascular events [75].

### 4.6 Effect of macrophages on renal function

Acute renal failure is a clinical critical disease characterized by a sharp decline in renal function, which is an important factor leading to an increase in the incidence rate of chronic kidney disease and ESRD worldwide. In renal failure caused by various diseases, the rapid recruitment and coordination of large numbers of monocytes and tissue resident macrophages is a key starting point for the occurrence and development of this disease. Macrophages play an important role in the human natural immune system, playing a role in immune defense, tissue remodeling, and maintaining dynamic balance of the body [76]. The mechanism of renal failure is complex and involves numerous pathways. Under the stimulation of infection or internal environment disorder, macrophages can be activated into a series of continuously controllable functional states, participating in processes such as improving the inflammatory microenvironment, inhibiting steatosis, promoting tissue repair, and anti-tumor immunity, namely, macrophage polarization [77,78,79]. After polarization, macrophages form M1/M2 phenotypes with mutually antagonistic functions. The former is activated by interferon and lipopolysaccharide and secretes a large amount of pro-inflammatory cytokines, which can promote pathogen clearance and inhibit tumor progression. The latter is activated by IL-4 and IL-13 and plays an anti-inflammatory and tissue repair promoting role. Macrophage polarization is crucial in the pathogenesis of renal failure. Based on our research results, we speculate that Macrophage inflammatory protein 1b is a negative regulatory transmembrane protein for macrophage inflammation, but there is currently no corresponding research to confirm this.

Unlike observational research, this study has unique advantages. This study mainly uses a large sample of genome-wide association research, which can better conduct a comprehensive analysis of different kidney disease events. However, this study has some limitations. The research object is limited to people of European origin. Although it can reduce the bias caused by population stratification, it cannot be proved that it applies to people of other races. As with all MR studies, this study cannot resolve the observed pleiotropy, so the results may be biased.

To sum up, this study shows that increased basophils count can increase the relative risk of chronic renal failure and renal tubular dysfunction, and reduce the risk of hypertensive heart disease and/or hypertensive nephropathy, while increased basophils count will not increase the relative risk of acute renal failure, increased neutrophils count can increase the risk of chronic renal failure, increased lymphocytes count can increase the relative risk of hypertensive nephropathy, and increased eosinophils count can increase the relative risk of type 1 diabetes with renal complications.
